# Lack of association between Triglyceride glucose-body mass index and glomerular filtration rate in patients with coronary heart disease after percutaneous coronary intervention: a retrospective case-control study

**DOI:** 10.3389/fendo.2026.1852416

**Published:** 2026-08-07

**Authors:** Xiaoxu Zhang, Zihe Zhang, Xiaoxiao Yu, Xiaoyuan Zhang, Haoyang Liu, Yina Le, Shan Wang, Feng Gao, Feng Yang, Jiahui Chen

**Affiliations:** 1 College of Rehabilitation Nursing, Hangzhou Polytechnic University, Hangzhou, China; 2 College of Second Clinical Medical College, Nanjing Medical University, Nanjing, China; 3 Department of Vascular Surgery, Hangzhou First People’s Hospital, Hangzhou, China; 4 College of The First Affiliated Hospital, Dalian Medical University, Dalian, China; 5 Department of Chronic Disease, Yipeng Community Health Service Center, Hangzhou, China; 6 Department of Clinical Nutrition, Hangzhou Cancer Hospital, Hangzhou, China

**Keywords:** chronic kidney disease (CKD), coronary heart disease (CHD), glomerular filtration rate (eGFR), percutaneous coronary intervention (PCI), Triglyceride glucose-body mass index (TyG-BMI)

## Abstract

**Background:**

There exists a close and complex bidirectional relationship between cardiovascular disease (CVD) and chronic kidney disease (CKD). CVD is a well-established risk factor for CKD, while the Triglyceride glucose-body mass index (TyG-BMI) has been considered as a predictor of cardiovascular events. This was a retrospective case-control study. This study aimed to investigate the relationship between preoperative TyG-BMI and estimated glomerular filtration rate(eGFR) during follow-up in patients with coronary heart disease (CHD) after percutaneous coronary intervention (PCI).

**Methods:**

This study was performed in 1020 patients with CHD after PCI without CKD from 01/01/2015 to 01/01/2025. We performed multifaceted analyses of the association between preoperative TyG-BMI and eGFR, including multiple linear regression for continuous eGFR outcomes, logistic regression to identify whether TyG-BMI independently influenced categorical eGFR status and quantify the magnitude of this association, and restricted cubic spline (RCS) analysis to explore non-linear relationships. Low eGFR (<60 mL/(min*1.73m²)) was set as the outcome variable, and was obtained from the latest follow-up.

**Results:**

The multivariate linear regression analysis revealed no statistically significant linear association between preoperative TyG-BMI and eGFR (*t* = -1.850, *P* = 0.065). Logistic regression indicated that TyG-BMI was not identified as an independent influencing factor of eGFR (*P* = 0.369, *OR* = 1.003, *95%CI:*0.977~1.009). RCS analysis indicated that the nonlinear association between TyG-BMI and eGFR was not statistically significant (*P* for nonlinear > 0.05).

**Conclusion:**

Preoperative TyG-BMI exerted no direct independent effect on long-term outcome of eGFR. It is unlikely that TyG-BMI serves as a primary determinant of CKD in patients with CHD after PCI. Future studies should evaluate combined risk models.

## 1 Introduction

Percutaneous coronary intervention (PCI) serves as a crucial therapeutic strategy for patients suffering from coronary heart disease (CHD), especially those with acute coronary syndrome (ACS) (1). The use of iodinated contrast agents and prolonged balloon angioplasty may result in iatrogenic acute kidney injury (PCI-AKI), with an incidence rate of 8.8% - 12.8% among patients undergoing PCI (2, 3). During surgical procedures, contrast agents have the potential to induce alterations in renal hemodynamics. Furthermore, contrast agents may demonstrate nephrotoxic properties and trigger the release of vasoactive substances, all of which can adversely impact renal function and potentially result in chronic kidney disease (CKD) (4). Currently, research has primarily focused on PCI-AKI. However, scant attention has been given to the occurrence and progression of CKD in patients who did not develop PCI-AKI at the time of PCI during long-term follow-up. Moreover, the combination of cardiovascular disease and PCI can easily lead to the development of cardiorenal syndrome (CRS) in patients.

Cardiovascular disease (CVD) and CKD often demonstrate a mutually deleterious relationship. In this dynamic, the two conditions interact with each other in such a way that they exacerbate one another, thereby leading to bidirectional harm and the establishment of a vicious cycle (5). The deterioration of cardiovascular system damage accelerates the decline in renal function, which, in a reciprocal manner, further affects the heart, ultimately creating a compounding effect of mutual harm (6). The onset of these two aforementioned diseases is associated with exposure to shared pathogenic factors, encompassing hypertension, dyslipidemia, aberrant glucose metabolism, obesity, and other cardiovascular metabolic risk elements (7). An increasing number of researchers are now turning to novel indicators that amalgamate multiple metabolic parameters for the prediction of CVD and CKD (8). Among the multitude of newly emerging metabolic markers, the triglyceride glucose-body mass index (TyG-BMI) stands out due to its strong correlation with both conditions (9). TyG-BMI is a composite index that combines measurements of insulin resistance (IR) and body mass index (BMI). It can serve as a surrogate indicator for assessing insulin resistance status.

Given the significance of understanding the interactions between metabolic and renal functions in patients with CHD after PCI, it is necessary to investigate the relationship between the TyG-BMI and estimated glomerular filtration rate (eGFR). This study designed a nested case - control study based on a retrospective cohort of patients with CHD after PCI. The aim was to evaluate how the comprehensive blood glucose and lipid metric, TyG-BMI, affects the crucial renal function indicator, eGFR, in patients following PCI.

## 2 Methods

### 2.1 Study design and population

**Figure 1:**
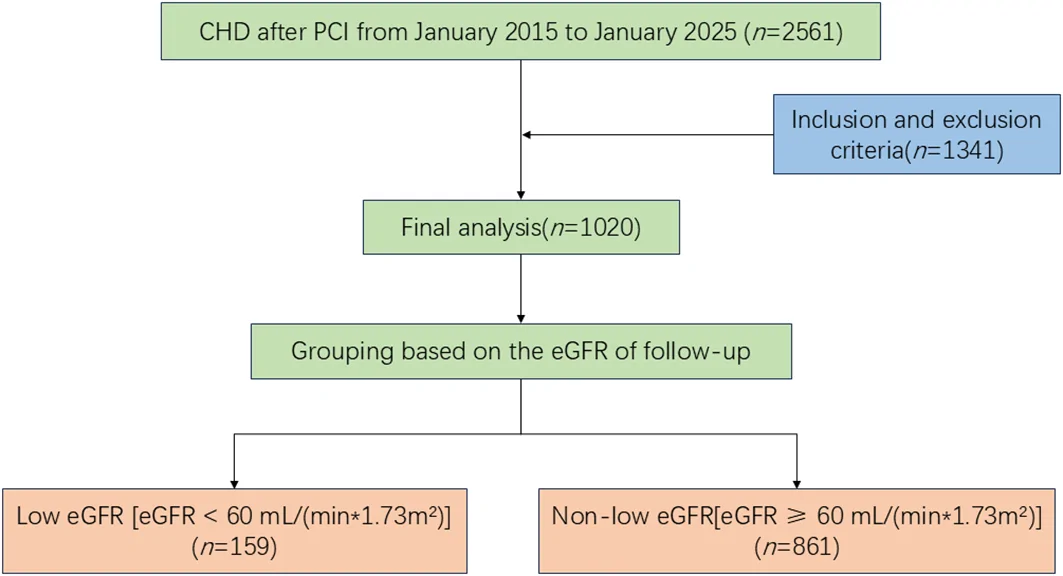
Flow chart.

This was a retrospective case-control study. The cases originated from the natural cohort of residents with CHD who underwent PCI were registered in Yipeng community health service center. The cohort mainly followed up and recorded the laboratory examination data related to hospitalization and physical examination of patients with CHD after PCI treatment. The cohort was jointly managed and maintained by general practitioners in chronic diseases from primary hospitals and professional data technicians in university. A 10-year retrospective period was set for this study. during a follow-up time of 3.0 (0.83-9.83) years. Through the resident health record data system, a total of 1020 patients with CHD who underwent PCI treatment (from January 2015 to January 2025) were included. The subjects were divided into two groups according to the eGFR obtained from their latest physical examination. Inclusion criteria: (1) Diagnosed with CHD and treated with PCI; (2) Arterial angiography review (3) Complete health record data. Exclusion criteria: (1) Cases of death in the past decade; (2) Cancer; (3) renal insufficiency; (4) Long-term medication for immune system diseases. This study followed the basic guidelines of the Declaration of Helsinki and was approved by the Ethics Committee. The data collection flow chart is shown in **Figure 1**.

### 2.2 Data collection and definitions

The health records of residents within the jurisdiction who met the inclusion and exclusion criteria were reviewed. Basic demographic information and relevant laboratory testing data of the research subjects were collected. All the baseline data were preoperative data for the PCI. All the research subjects received dual antiplatelet therapy with oral aspirin 100mg/day and clopidogrel 75mg/day or ticagrelor 90mg/day for at least 1 year after PCI. The research subjects with chronic diseases such as diabetes and hypertension were treated with medicine according to the doctor’s advice.

Since this was an observational cohort, the subjects were not required to undergo a physical examination annually. As a result, the frequency of physical examinations was not fixed at once a year. The eGFR data were obtained from the latest follow-up before data retrieval, and values below 60 mL/(min*1.73m²) were classified as low eGFR (10). The eGFR was calculated as follows:

The estimated glomerular filtration rate (eGFR) (ml/min/1.73m^2^) was calculated using the CKD-EPI formula: Male: serum creatinine ≤ 0.9ml/dl: eGFR=141×(serum creatinine/0.9)^-0.411^×(0.993)^age^; Serum creatinine > 0.9 ml/dl: eGFR = 141 × (serum creatinine/0.9)^-1.209^×(0.993)^age^. Female: Serum creatinine ≤ 0.7ml/dl: eGFR=144×(serum creatinine/0.7)^-0.329^×(0.993)^age^; Serum creatinine >0.7ml/dl: eGFR=144×(serum creatinine/0.7)-^1.209^×(0.993)^age^ (11).

Based on the interquartile range (IQR) of TyG-BMI values, study participants were stratified into four groups. The TyG, BMI and TyG-BMI was calculated as follows:


$$\mathrm{body}\;\mathrm{mass}\;\mathrm{index}\;\left(\mathrm{BMI}\right):\;\mathrm{BMI}=\;\mathrm{weight}\;\left(\mathrm{kg}\right)/\mathrm{height}\;{\left(\text{m}\right)}^{2}$$

TyG=ln[TG(mg/dl)×FBG(mg/dl)/2]

(12)


TyG−BMI= ln[TG(mg/dl)×FBG(mg/dl)/2]×BMI

### 2.3 Statistical analysis

The data for this study were organized via Excel software, and the data analysis was conducted via SPSS 25.0 software and R4.6.0. Numerical data that followed a normal distribution were presented as the means ± standard deviation, and *t*-tests were used to compare the means between groups. Numerical data that did not follow a normal distribution were presented as medians (25%quantile,75%quantile), and the nonparametric rank sum test was used to compare the medians among groups. Categorical data were presented as absolute values (percentages), and the chi-square test was used to compare proportions between groups. When the categorical data did not meet the assumptions for the chi-square test, Fisher’s exact test was used instead. It conducted a multifaceted analysis of the relationship between exposure TyG-BMI and outcome eGFR by implementing multiple liner regression to model continuous outcomes. Logistic regression was performed to identify whether TyG-BMI independently influenced categorical eGFR status and to quantify the magnitude of this association. And restricted cubic spline (RCS) analysis to detect and characterize non-linear relationships.

## 3 Results

### 3.1 The relationship between preoperative data and eGFR of follow-up

A total of 1020 participants with PCI were included. Mean age was 62.31 ± 10.53 years, with 521 (51.1%) males. Low eGFR was defined as eGFR< 60 mL/(min*1.73m²). Among them, 159 cases (15.6%) exhibited low eGFR.

Age, sex, Follow-up, HBP, BNP, cTnT, Plt, and FIB showed statistically significant differences between the two groups in terms of whether they had low eGFR(*P*<0.05), while no statistically significant differences were observed for the other variables between the two groups(*P*>0.05), see **Table 1** for details.

**Table 1:** Correlation analysis between preoperative data and low eGFR.

Variables	Total (*n* = 1020)	Low eGFR		*t/χ^2^/z*	*P*
		No (*n* = 861)	Yes (*n* = 159)		
Age (years)	62.31 ± 10.53	61.02 ± 10.14	69.18 ± 10.59	-9.279	**0.001**
Sex (%)					
Men	499 (48.9)	462 (92.6)	37 (7.4)	49.599	**0.001**
Women	521 (51.1)	339 (76.6)	122 (23.4)		
HBP (%)					
No	322 (31.6)	294 (91.3)	28 (8.7)	16.989	**0.001**
Yes	698 (68.4)	567 (81.2)	131 (18.8)		
MD (%)					
No	649 (63.6)	556 (85.7)	93 (14.3)	2.148	0.143
Yes	371 (36.4)	305 (82.2)	66 (17.8)		
Stroke (%)					
No	928 (91.0)	787 (84.8)	141 (15.2)	1.215	0.270
Yes	92 (9.0)	74 (80.4)	18 (19.6)		
Smoking(%)					
No	678 (66.5)	566 (83.5)	112 (16.5)	1.332	0.248
Yes	342 (33.5)	295 (86.3)	47 (13.7)		
Drinking (%)					
No	761 (74.6)	633 (83.2)	128 (16.8)	3.456	0.063
Yes	259 (25.4)	228 (88.0)	31 (12.0)		
Follow-up (years)	3.66 ± 2.41	3.53 ± 2.31	4.33 ± 2.79	-3.405	**0.001**
FBG	6.51 ± 2.70	6.42 ± 2.61	6.82 ± 2.97	-1.723	0.085
TC (mmol/L)	3.35 ± 1.69	3.35 ± 1.73	3.31 ± 0.85	0.230	0.818
TG (mmol/L)	1.45 ± 0.83	1.45 ± 0.87	1.43 ± 0.64	0.227	0.820
HDL-C (mmol/L)	1.08 ± 0.32	1.07 ± 0.32	1.07 ± 0.31	0.342	0.732
LDL-C (mmol/L)	1.85 ± 0.69	1.85 ± 0.69	1.85 ± 0.68	-0.046	0.963
BNP (pg/ml)	87.00 (40.6, 156.70)	82.40 (38.10,142.15)	128.00 (70.60,261.00)	-6.680	**0.001**
cTnT (μg/L)	0.01 (0.01,0.02)	0.01 (0.01,0.02)	0.01 (0.02,0.04)	-8.805	**0.001**
Plt (109/L)x	201.33 ± 52.56	203.44 ± 51.73	186.73 ± 52.39	3.735	**0.001**
FIB (mg/dL)	3.09 ± 0.73	3.04 ± 0.67	3.29 ± 0.98	-4.025	**0.001**
Lp-a (mmol/L)	16.04 (6.99,41.32)	16.58 (7.46,41.32)	13.18 (5.20,41.32)	-1.784	0.074
D-D (ug/ml)	0.27 (0.21,0.41)	0.25 (0.20,0.37)	0.39 (0.26,0.81)	-8.122	**0.001**
APTT(s)	35.36 ± 6.04	35.25 ± 5.36	35.99 ± 8.47	-1.450	0.147

Data are presented as mean ± standard deviation, median (interquartile range) or percentage, as appropriate. Values in bold indicate statistically significant differences (P < 0.05).

### 3.2 The relationship between TyG-BMI data and eGFR of follow-up

**Figure 2:**
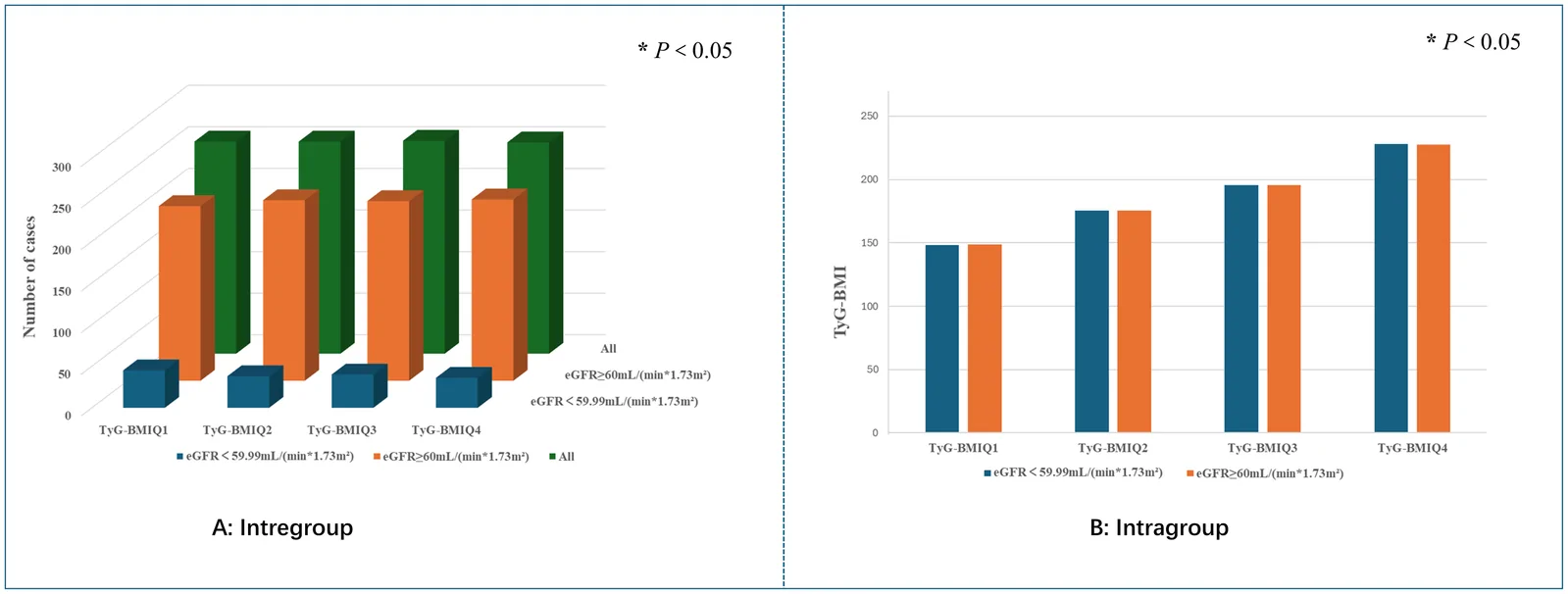
Correlation analysis between TyG-BMI data and low eGFR.

No statistically significant difference in TyG, BMI, and TyG-BMI (different forms) was observed between the low eGFR and non-low eGFR groups (*P*>0.05). See **Table 2** and **Figure 2** for details.

**Table 2:** Correlation analysis between TyG-BMI data and low eGFR (*n* = 1020).

Variables	Total (*n* = 1020)	Low eGFR		*t*	*P*
		No(*n* = 861)	Yes(*n* = 159)		
TyG	7.14 ± 0.63	7.13 ± 0.63	7.19 ± 0.65	-1.131	0.259
BMI	26.09 ± 3.05	26.15 ± 3.33	25.77 ± 3.77	1.199	0.232
TyG-BMI	186.78 ± 32.05	186.96 ± 31.70	185.84 ± 34.25	0.383	0.702

### 3.3 The relationship between TyG-BMI and eGFR of follow-up with multivariate analysis

#### 3.3.1 Multiple linear regression analysis

eGFR was treated as a continuous variable, and multiple linear regression was applied to assess the relationship between TyG-BMI and eGFR. eGFR was set as the dependent variable; independent variables consisted of variables with *P* values less than 0.05 in **Table 1** and the key variable TyG-BMI. Variable selection was performed using the backward elimination method, with a significance threshold of 0.05 for variable inclusion and 0.1 for exclusion. The analysis confirmed the absence of multicollinearity among the independent variables, as all variance inflation factors (VIFs) were below 10. The results showed that there was no linear correlation between TyG-BMI and eGFR (*P*<0.05). See **Table 3** for details.

**Table 3:** Multiple linear regression analysis.

Variables	B	SE	*β*	*t*	*P*
Age	-0.499	0.056	-0.255	-8.973	**0.001**
Sex	-16.276	1.047	-0.392	-15.539	**0.001**
HBP	-0.018	0.004	-0.113	-4.254	**0.001**
Plt	0.048	0.010	0.123	4.678	**0.001**
D-D	-9.973	1.656	-0.164	-6.021	**0.001**
TyG-BMI	-0.032	0.017	-0.049	-1.850	0.065

Values in bold indicate statistically significant differences (P < 0.05).

#### 3.3.2 Logistic regression analysis

**Figure 3:**
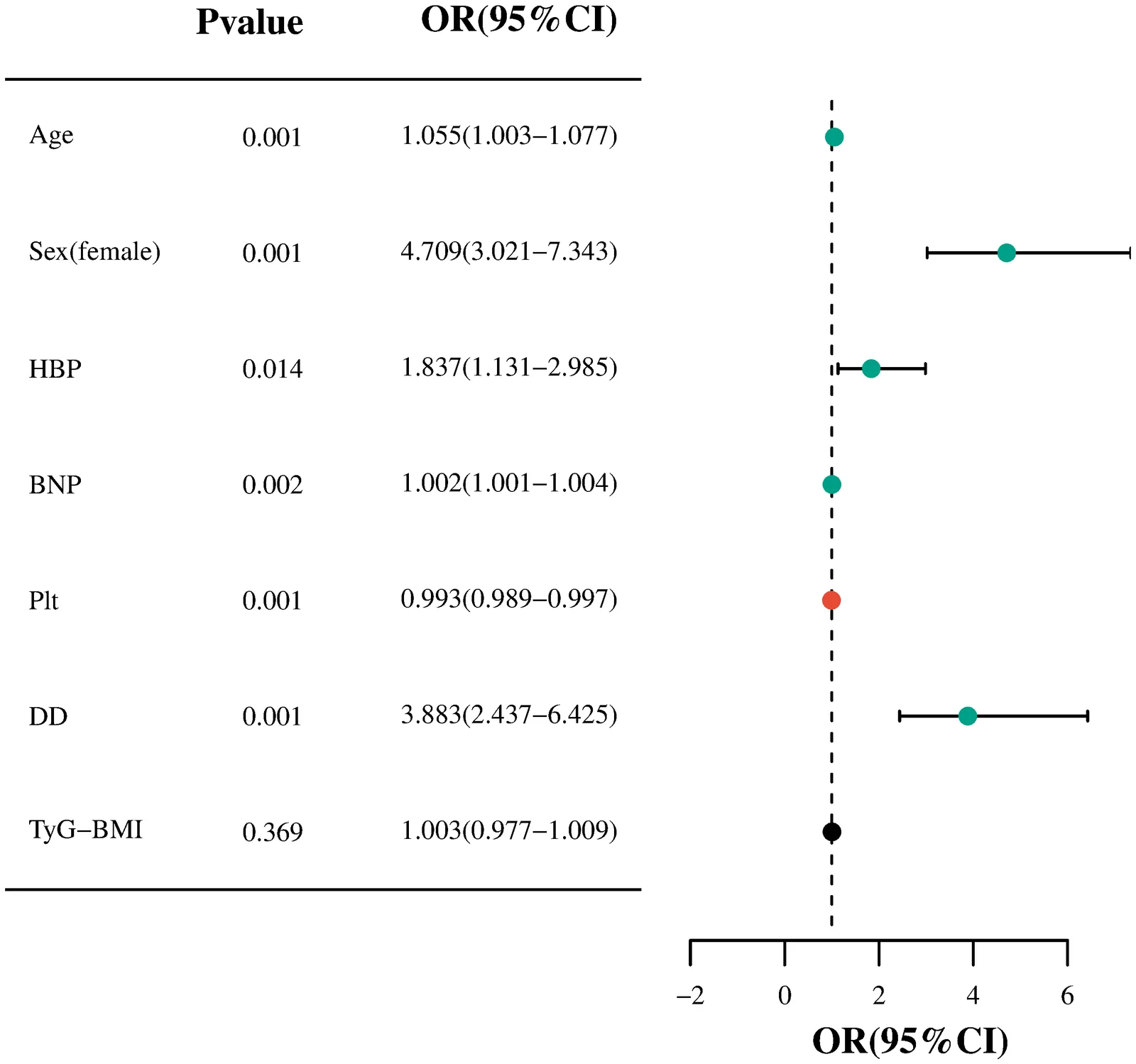
Logistic regression analysis.

A logistic regression model was constructed with binary variable eGFR as the outcome. After adjusting for confounding factors, we analyzed whether independent variable TyG-BMI was an independent influencing factor for the occurrence of low eGFR, and the odds ratio (OR) was used to quantify the strength of association between TyG-BMI and eGFR. eGFR was set as the dependent variable; independent variables consisted of variables with P values less than 0.05 in **Table 1** and the key variable TyG-BMI. Variable selection was performed using the backward elimination method, with a significance threshold of 0.05 for variable inclusion and 0.1 for exclusion. After adjustment for confounders, TyG-BMI was not identified as an independent influencing factor of eGFR (*P* = 0.369, *OR* = 1.003, *95%CI:*0.977~1.009). See **Figure 3** for details.

#### 3.3.3 RCS analysis

**Figure 4:**
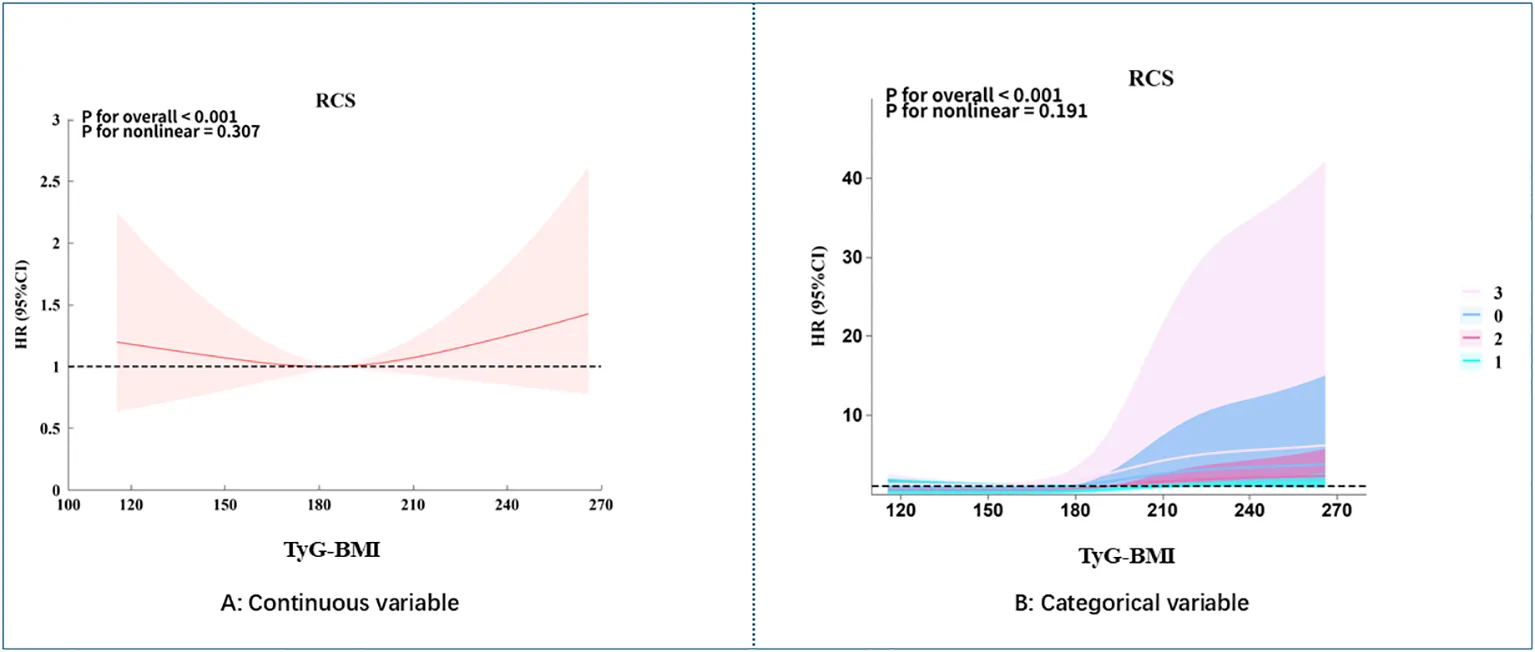
RCS analysis.

In order to better understand the impact of TyG - BMI on eGFR, we examined the nonlinear association between TyG-BMI and eGFR using RCS visualization. The analysis showed that the nonlinear association between TyG-BMI and eGFR was not statistically significant (*P* for nonlinear>0.05), regardless of whether TyG-BMI was modeled as a continuous or categorical variable. An associative trend was observed between TyG-BMI and eGFR(*P* for overall<0.05); however, the prior multiple linear regression model did not identify a statistically significant linear association(*P*>0.05), which may be attributable to interval superposition effects. See **Figure 4** for details.

### 3.4 Sensitivity analysis

To verify the robustness of the primary analysis results, a series of sensitivity analyses were performed based on different outcome definitions, follow-up durations and subgroup populations. When we separately adopted four analytical settings: the outcome variable was defined as the average of baseline and last follow-up eGFR (Model 1), patients with follow-up duration less than 2 years were excluded (Model 2), the study population was restricted to non-hypertensive individuals (Model 3), and eGFR< 40 mL/(min*1.73 m²) was set as the outcome (Model 4), we observed no statistically significant associations across all sensitivity models (*P* > 0.05), which were consistent with the conclusions of the main model. All sensitivity analyses did not alter the negative result of the main model, indicating that this association has good robustness under different settings. See **Table 4** for details.

**Table 4:** Sensitivity analysis.

Model	Linear [*t*(*P*)]	Logistic [*Wald*(*P*)]	RCS (*P*/*P*)*
Main model	-1.805 (0.065)	0.982 (0.322)	**<0.001** (0.367)
Model 1	-1.469 (0.142)	1.096 (0.295)	0.156 (0.421)
Model 2	1.037 (0.300)	0.285 (0.594)	0.338 (0.579)
Model 3	-0.560 (0.576)	0.033 (0.856)	0.462 (0.485)
Model 4	-1.805 (0.065)	0.491 (0.483)	0.071 (0.403)

Model 1: The outcome variable was defined as the average of the baseline and last follow-up eGFR values.

Model 2: Patients with a follow-up duration of less than 2 years were excluded. The sample size was 694.

Model 3: Patients without hypertension. The sample size was 322.

Model 4: The outcome variable was defined as <40 mL/(min*1.73m²).Cases of Low eGFR was 39.

All covariates were consistent with those in the main model.

*First P=P for overall, Second P=P for nonlinear. Values in bold indicate statistically significant differences (P < 0.05).

## 4 Discussion

This study focused on patients with CHD who have undergone PCI, aiming to investigate the association between the preoperative TyG-BMI measurements and the follow-up eGFR. A 10-year follow-up review showed that 15.6% of the patients with CHD who had undergone PCI had experienced a reduced eGFR (mL/(min*1.73m²)). The research findings indicated that TyG-BMI has no direct, independent effect on the follow-up eGFR(*P*>0.05). Notably, this conclusion remains valid even after adjustments had been made for other clinical confounding variables. These results suggest that TyG-BMI is unlikely to act as a key determinant in the changes of renal function among patients with CHD undergoing PCI during the follow-up period.

Many previous studies suggested that CKD was an important factor affecting the progression and adverse outcomes of CHD, whereas this study focused on the impact of CHD on CKD (13). A retrospective study conducted between 2010 and 2019 included 359 CHD patients. Those with an eGFR< 90 mL/(min/1.73m²) were diagnosed as having CKD. Among these patients, 167 (46.5%) developed CKD (14). A cross-sectional study, which utilized data from the China Heart Survey (CHS), revealed that among the 3,513 CHS participants diagnosed with CHD, the prevalence of CKD was 24.8%(n=871) (15). Their proportion values were higher than the 15.6% that had been reported in our study. An observational and prospective cohort study in which a total of 657 adult CHD patients were enrolled, showed that the prevalence of CKD (defined as a Glomerular filtration rate (GFR)< 60 mL/min/1.73 m²) was 0.2% during a follow-up time of 6.8 (1.2-10.5) years (16). This proportion was lower than the 15.6% reported in our study. A study investigated the prevalence of CKD in CHD patients from 24 European countries. A total of 7,998 participants (median 65 years of age, 76% male) attended a study visit 6–36 months after the index hospitalization. During the follow-up, 17.3% of them had CKD (eGFR< 60 mL/min/1.73 m²) (17). This proportion was similar to obtained in our research. A large cross-sectional epidemiologic study, which collected data from 5,942 men and women aged 30 to 69 years showed that CKD was a major risk factor for the development of ischemic heart disease (18). Incident hospitalization with cardiac diseases (i.e. HF and CHD) was significantly associated with a subsequent acceleration of eGFR decline (19).

A review of previously published related research revealed that there is a certain correlation between metabolic indicators and renal function. However, the outcomes of our study diverged from those reported in these preceding investigations. In a study involving 5484 non-diabetic patients, the correlation between TyG and CKD was investigated. Compared with participants in the lowest TyG quartile, in the fully adjusted models (which adjusted for factors such as age, gender, and so on), the hazard ratio (HR) and 95% confidence interval (CI) of incident CKD were 1.772 (*95% CI:* 1.453~2.162) for those in the highest TyG index variability quartile and 2.091 (*95% CI:* 1.646~2.655) for those in the highest cumulative TyG index quartile, respectively (20). Another retrospective study showed that the relationship between TyG index and CKD was non-linear (*P* non-linearity=0.021) (21). However, there existed studies that shared similarities with our study; for instance, a Chinese study demonstrated that after adjusting for confounders, TyG-WHtR and TyG-WC remained significantly associated with CKD, while TyG-BMI did not (22). The discrepancy in findings could potentially stem from variations in the study populations. Prior research might have encompassed a broader range of patient groups, including those with different types of CVD as well as patients at various stages of the disease (23, 24). In contrast, this study specifically targeted patients with CHD who underwent PCI treatment (25). This group of patients showed distinct disease traits, treatment modalities, and metabolic profiles, which could have accounted for the differing impacts of TyG-BMI on eGFR. In addition, differences in research methods may also have been factors leading to inconsistent results. Different studies may have had differences in inclusion and exclusion criteria, adjustment range for confounding factors, and follow-up time and methods. This study implemented stringent inclusion and exclusion screening criteria for specific populations, and comprehensively adjusted for clinically pertinent confounding factors from multiple dimensions through the application of diverse statistical methods. Concurrently, the longitudinal nature of the data collected in this research took full account of the influence of temporal effects on outcomes (26). The aforementioned study design facilitated an objective and accurate assessment of the relationship between TyG-BMI and eGFR in CHD patients after PCI.

The results of this study have certain exclusion value for clinical practice. Given that TyG-BMI lacks independent predictive power for assessing the long-term renal function of patients with CHD after PCI, general practitioners should avoid relying too much on it as an indicator when evaluating the risk of CKD in such patients (27). Instead, proactive consideration should be given to utilizing other, more effective risk stratification tools for predicting the risk of renal function decline. A comprehensive risk scoring model based on multiple clinical indicators and biomarkers may be able to more comprehensively reflect the overall health status and potential risks to renal function of patients (28, 29), helping clinicians to more accurately identify potential renal function decline patients in CHD patients after PCI, and thus develop personalized treatment and follow-up strategies.

## 5 Conclusions

Preoperative TyG-BMI showed no direct independent effect on long-term eGFR outcomes in this study. After adjustment for confounding factors, it did not independently predict long-term renal function change. These results indicate that TyG-BMI is unlikely to be a primary determinant of CKD in CHD patients after PCI. Thus, general practitioners and clinicians should not rely solely on TyG-BMI for renal risk stratification. Instead, more reliable and comprehensive risk assessment tools are recommended for evaluating long-term renal risk in this patient population.

Notably, TyG-BMI exhibited a borderline *P* value of 0.065 in Multiple linear regression analysis, indicating a weak marginal trend. However, the observed standardized mean difference (Cohen’s *d* = 0.035) revealed an extremely trivial intergroup disparity, and power analysis confirmed our 1020 participants’ cohort was adequately powered to detect clinically meaningful differences. Although a substantially larger sample may render this weak trend statistically significant, such a minimal difference carries limited clinical interpretive value. Future large-scale multicenter cohorts are warranted to further verify this subtle marginal signal.

## 6 Limitations

Although all participants were community-managed CHD patients and excluding subjects with acute diseases, AKI effects could not be completely eliminated. Low eGFR was determined by a single final follow-up test instead of two tests spaced ≥3 months as standard CKD diagnosis demands. Our findings merely reflect cross-sectional correlations with renal function rather than associations with confirmed CKD. Prospective studies with serial eGFR measurements are required to verify these conclusions. In addition, there was heterogeneity in follow-up duration (0.83–9.83 years). Although consistent results were obtained in sensitivity analyses, which alleviated the impact of detection bias, our findings only reflect associations within the current observation period, and further studies with standardized follow-up are needed to verify our conclusions.

## Data Availability

The data analyzed in this study is subject to the following licenses/restrictions: The data owned by a third-party organization: Qiantang District Center for Disease Control and Prevention. Requests to access these datasets should be directed to Qiantang District Center for Disease Control and Prevention via email (qtwjw@mail.gov.cn).
